# A microbial carbonate response in synchrony with the end-Triassic mass extinction across the SW UK

**DOI:** 10.1038/srep19808

**Published:** 2016-01-27

**Authors:** Yadira Ibarra, Frank A. Corsetti, Sarah E. Greene, David J. Bottjer

**Affiliations:** 1 University of Southern California, Department of Earth Sciences, 3651 Trousdale Parkway, Los Angeles, California 90089 USA; 2 Stanford University, Department of Earth System Science, 450 Serra Mall, Bldg 320, Stanford, CA 94305 USA; 3 School of Geographical Sciences, University of Bristol, University Road, Bristol BS8 1SS, UK

## Abstract

The eruption of the Central Atlantic Magmatic Province (CAMP)—the largest igneous province known—has been linked to the end-Triassic mass extinction event, however reconciling the response of the biosphere (at local and nonlocal scales) to potential CAMP-induced geochemical excursions has remained challenging. Here we present a combined sedimentary and biological response to an ecosystem collapse in Triassic-Jurassic strata of the southwest United Kingdom (SW UK) expressed as widely distributed carbonate microbialites and associated biogeochemical facies. The microbialites (1) occur at the same stratigraphic level as the mass extinction extinction, (2) host a negative isotope excursion in δ^13^C_org_ found in other successions around the world, and (3) co-occur with an acme of prasinophyte algae ‘disaster taxa’ also dominant in Triassic-Jurassic boundary strata of other European sections. Although the duration of microbialite deposition is uncertain, it is likely that they formed rapidly (perhaps fewer than ten thousand years), thus providing a high-resolution glimpse into the initial carbon isotopic perturbation coincident with the end-Triassic mass extinction. These findings indicate microbialites from the SW UK capture a nonlocal biosedimentary response to the cascading effects of massive volcanism and add to the current understanding of paleoecology in the aftermath of the end-Triassic extinction.

The end-Triassic extinction, one of the ‘big five’ mass extinctions of the Phanerozoic^1^, has been linked to the eruption of the Central Atlantic Magmatic Province (CAMP)^2^. High atmospheric *p*CO_2_^3^,^4^,^5^ and the release of volcanic volatiles via massive CAMP eruptions likely resulted in widespread warming and the spread of anoxia in shallow marine environments^6^,^7^ and may have contributed to the collapse of terrestrial and marine ecosystems. The extinction is coincident with the so-called “initial carbon isotope excursion” (I-CIE), a negative δ^13^C_org_ excursion in marine and terrestrial strata considered a global chemostratigraphic marker^8^, and in European sections, the extinction coincides with a bloom of prasinophyte algae^6^,^9^,^10^ considered “disaster taxa”^11^.

Across the southwestern United Kingdom (SW UK), the end-Triassic mass extinction interval contains a considerably widespread (>2,000 km^2^) microbialite-bearing carbonate unit known regionally as the ‘Cotham Marble’^12^,^13^,^14^ whose distinct morphology and geologic significance has been the subject of much speculation in the scientific literature since the 18^th^ century^15^. We present combined biogeochemical and petrographic evidence that indicates the Cotham Marble microbialites host geochemical (I-CIE) and biological characteristics (abundant fossilized clusters of prasinophyte algae) that directly link them to other UK and European end-Triassic sections. The tightly coupled sedimentary, biological, and chemical response captured by the microbialites reflects the crucial but often overlooked role of microbial carbonates as sedimentary archives during times of environmental crisis.

## Regional Setting

**Figure 1:**
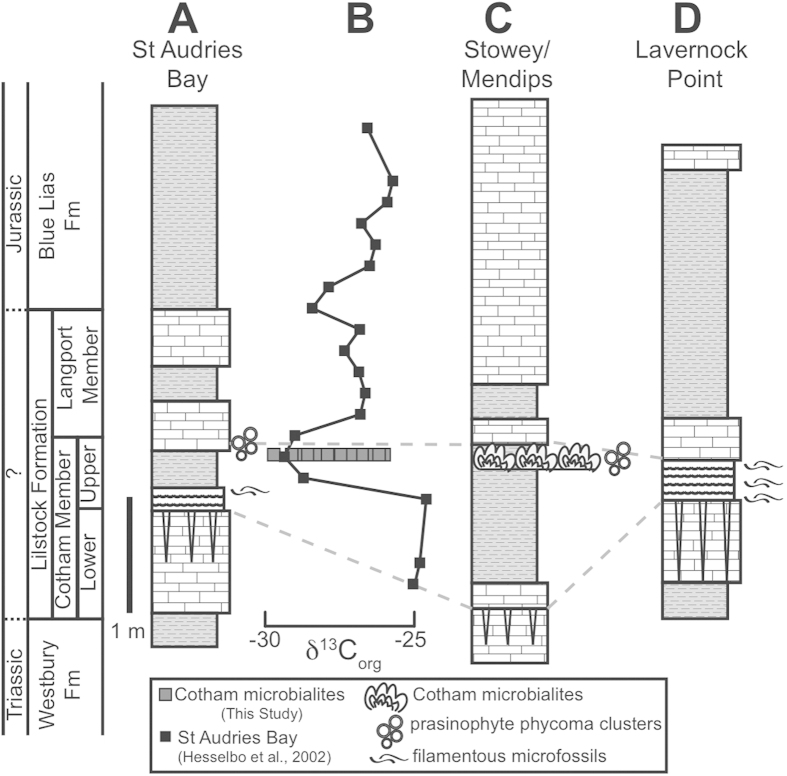
Upper Triassic stratigraphy of the Southwestern United Kingdom. (**A**) Generalized stratigraphic column from St Audrie’s Bay. (**B**) δ^13^C_org_ from St Audrie’s Bay^8^ and microbialite δ^13^C_org_ data from this study. (**C**) Stratigraphic column from Stowey Quarry and the Mendips region (modified from ref. 21). (**D**) Stratigraphic column from Lavernock Point.

Uppermost Triassic to lowermost Jurassic strata of the SW UK, represented by the Westbury, Lilstock, and Blue Lias Formations, were deposited in shallow epicontinental seaways connected to the Tethys Ocean to the south^16^. The sections around the St Audrie’s Bay area are arguably the best studied, where the extinction horizon is found within the Upper Cotham Member of the Lilstock Formation^17^, above a fissured interval (interpreted by some as a subaerial exposure surface) that separates the Lower Cotham Member from the Upper Cotham Member (Fig. 1). At St Audrie’s Bay, the Upper Cotham Member contains the I-CIE coincident with the extinction horizon in many sections around the world. The position of the extinction horizon in the SW UK is debated due to the coincidence of the extinction and paleoenvironmental change (shallowing), but is generally presumed to occur at or near the I-CIE^17^,^18^.

**Figure 2:**
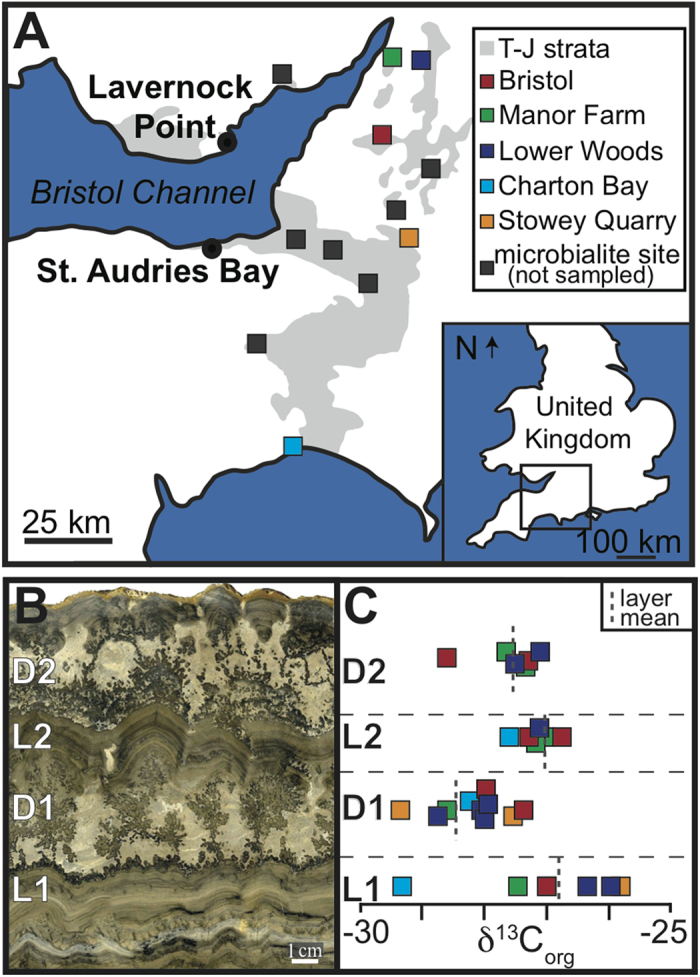
Regional Map (modified from ref.^17^). (**A**) Study sites and other Cotham Marble microbialite localities. (**B**) Laminated (L) and dendrolitic (D) clyclic mesostructure of the Cotham Marble microbialites (sample location: Bristol). (**C**) δ^13^C_org_ of four of the Cotham Marble microbialite phases by location.

Of note, the Upper Cotham Member contains meter-scale microbialite mounds that crop out discontinuously for over 2,000 km^2^ (Fig. 2A)^13^,^19^. The microbialites occur about a meter above the fissured horizon at the top of the Upper Cotham Member. Although they are widespread, the microbialite mounds do not occur in all localities where the Upper Cotham Member is present. In particular, they are absent from the well-studied sections at St Audrie’s Bay and Lavernock Point, rendering the stratigraphic position of the microbialite mounds with respect to the extinction and carbon isotopic excursion unclear (until now).

## Results

Despite the distance between sampling sites (over 100 km in some cases) and the discontinuous nature of the microbialite mounds, the samples studied display a strikingly persistent cyclic mesomorphology^13^,^19^, consisting of alternating stromatolitic intervals and dendrolitic intervals (L1, D1, L2, D2, Fig. 2B). Close inspection at the sub mm-scale reveals the alternations between laminated and dendrolitic intervals are essentially identical from site to site^19^ although variability in mesomorphology is common^20^.

Carbon isotope analyses of the bulk organic carbon within the microbialite unit from four different localities reveal values ranging from −25.8‰ to −29.6‰ (Fig. 2 and Table S1). The overall range in values spans most of the I-CIE measured at St Audrie’s Bay and occurs at the same stratigraphic level (Fig. 1) based on the most likely correlations between microbialite-bearing sites and St Audrie’s Bay^21^. For most studied microbialites, the most negative δ^13^C_org_ values occur in the first dendrolitic phase (D1), a pattern observed in sites around Bristol and further afield in Charton Bay (Fig. 2C). No correlation was found between paired inorganic carbon and oxygen isotopic values (Fig. S1 and Table S2), suggesting a lack of an evaporative effect due to restriction^22^.

**Figure 3:**
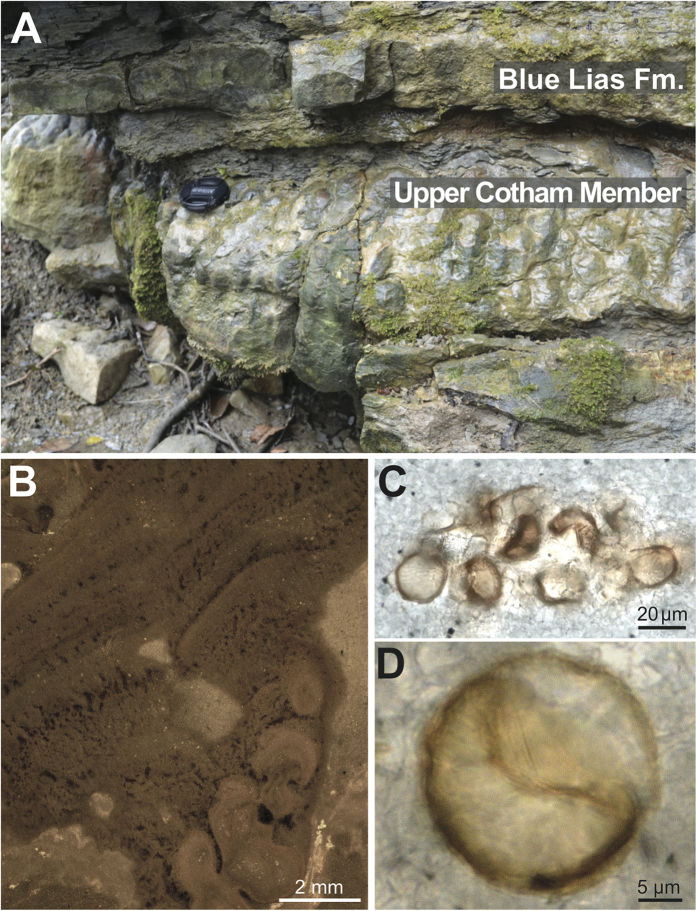
Cotham Marble microbialite facies. (**A**) Outcrop photograph of the microbialites from Lower Woods Natural Reserve. (**B**) Dendrolitic and stromatolitic couplet of the microbialites (Manor Farm). (**C**) Thin section photomicrograph of a cluster of round organic-walled microfossils from St Audrie’s Bay. (**D**) Close-up of a single organic-walled microfossil displaying a diagonal suture (Manor Farm).

Petrographically, the microbialites display intricate dendritic branching patterns and well-preserved fine-grained stromatolitic laminae (Fig. 3). The interstitial fill between the dendrolitic microbialite fabric contains distinct clusters of spherical, organic-walled cells (~10–100 μm in diameter) that were detected in microbialites from all of the sites investigated and also occur in clusters in the Upper Cotham Member at St Audrie’s Bay (Fig. 3C,D). Common features of the organic-walled cells are linear diagonal sutures and radially-arranged wall canals (Fig. 3D and Fig. S2) identified as prasinophyte phycomata assignable to the green alga *Tasmanites* (H. Agic, pers. comm.).

**Figure 4:**
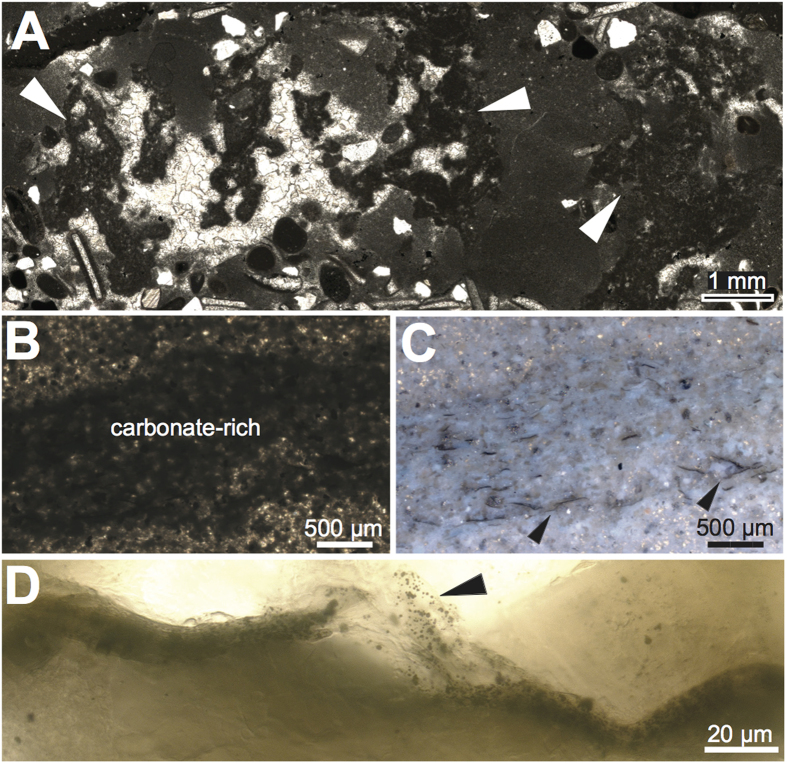
Microfacies of the Upper Cotham Member from Lavernock Point, South Wales. (**A**) Micritic dendrites from the oolitic bed (denoted by white arrows). (**B**) Dark carbonate-rich forset lamination under transmitted light. (**C**) Same field of view as Fig. 4A under reflected light with arrows denoting filamentous microfossils. (**D**) Close-up of filamentous microfossil.

Although the microbialites do not occur at St Audrie’s Bay and Lavernock Point, perhaps due to a greater component of clastic input, mm-scale dendrolitic fabrics reminiscent of the microbialite dendrolitic phases (Fig. 4A) occur in the oolitic beds above the fissured horizon at Lavernock Point suggesting a connection to the microbialite dendrolitic phases and corroborating our regional correlations. In addition, the shallow rippled facies of the Upper Cotham Member in St Audrie’s Bay and the correlative heterolithic facies of Lavernock Point contain copious filamentous microfossils (~10 μm in diameter and ~100–200 μm in length) (Fig. 4B,C). The filaments are aligned parallel to the foreset laminae of ripple marks and are associated with sedimentary pyrite, quartz, and carbonate-rich laminations (Fig. 4B–D and Figs. S3–S4).

## Discussion

Several lines of evidence converge to suggest the microbialites of the Upper Cotham Member were coincident with the end-Triassic extinction:

1. The shift in δ^13^C_org_ from about −25.8‰ to −29.6‰ within the microbialites (Figs. 1 and 2C) corresponds in absolute value and magnitude to the I-CIE from St Audrie’s Bay^8^, where the I-CIE co-occurs with the extinction^17^ and has been correlated with the I-CIE at other European sites, where it also co-occurs with the extinction^10^. Additionally, the equivalence of the I-CIE and the Cotham Marble microbialites has been demonstrated at Stowey Quarry^21^.
2. The microbial features (filamentous microfossils and organic-walled spherical microfossils) occur at the same horizon as the I-CIE at St Audrie’s Bay. The presence of similar dendrolitic carbonate forms at the extinction horizon at Lavernock Point, is suggestive of more direct correlation. When combined with the isotopic and microfossil data, the case becomes compelling.
3. Acmes of prasinophytes have been observed across the I-CIE in other Triassic-Jurassic sections in Europe^9^,^10^ and across the Triassic-Jurassic interval in Panthalassa^23^. In the United Kingdom, the first dendrolitic phase (D1) of the microbialites, which contains the I-CIE (Fig. 2C), hosts the prasinophyte phycomata acme^13^ and a second ‘main carbon isotope excursion’ measured at St Audrie’s Bay also coincides with a bloom of prasinophyte phycomata assignable to *Tasmanites*^24^.
4. The striking similarity at the meso-micro scale between microbialites 100 km apart suggest they grew synchronously and in response to non-local forcing, likely associated with forcings related to the mass extinction.

Thus, multiple lines of evidence would suggest the microbialites of the Upper Cotham Member are indeed coincident with the end-Triassic extinction event. As finely laminated structures, the microbialites may offer an unprecedented higher resolution glimpse into the extinction and thus could provide additional insight into mechanisms and effects of the extinction. It is not clear how rapidly stromatolites or dendrolites accrete, but typical estimates would suggest lamina could represent diurnal, seasonal, yearly, or perhaps multi-year^25^ timescales. Thus, it is unlikely that the microbialite mounds could represent more than “thousands” of years of deposition (that is, it is unlikely that the microbialites represent tens or hundreds of thousands of years). This is particularly true for the Cotham Marble microbialites, which preserve very delicate structures that would require rapid lithification. Considering the mean isotopic composition of each layer, the data can be interpreted to represent the initiation of the I-CIE, giving an unprecedentedly high-resolution glimpse into the early stages of the I-CIE provided by the geologically rapid deposition of the Cotham Marble microbialites.

The I-CIE is taken by some authors^26^ to indicate an input of isotopically light δ^13^C, which isotopically depleted the global atmospheric and ocean carbon reservoirs. There is some support for this interpretation by reports of excursions in δ^13^C_carb_ at many sites globally, presumed to reflect an isotopically depleted DIC pool; however this is debated^27^,^28^. The δ^13^C_carb_ from the Cotham Marble microbialites, however, do not show a negative excursion coincident with the excursion in δ^13^C_org_ (Fig. S1). Nonetheless, an excursion in δ^13^C_org_ co-occurring with stasis in δ^13^C_carb_ is entirely reconcilable. If CAMP emplacement injected mantle CO_2_ (which has a similar carbon isotopic composition to riverine weathering input) into the atmosphere-ocean system, the bulk ocean inorganic δ^13^C signature may be little affected (yielding stasis in δ^13^C_carb_), while the fractionation factor associated with organic carbon formation is increased due to increasing atmospheric CO_2_ concentrations (resulting in a negative excursion in δ^13^C_org_) 29. A second possibility is that the I-CIE in C_org_ simply reflects a shift in the composition of bulk organic matter. The occurrence of the I-CIE at the same level as peaks in green algae in the microbialites and in other end-Triassic sections^6^,^9^,^10^ is striking, suggesting a possible causal relationship, whereby the CIE in bulk organic matter is the result of organic matter compositional changes rather than a global shift in δ^13^C_DIC_ or C_org_ fractionation factor. Indeed, although the I-CIE is commonly used as a chemostratigraphic marker for the end-Triassic extinction, the connection between carbon isotope excursions and a perturbation to the global carbon cycle across the Triassic-Jurassic boundary has not been clearly established^27^,^28^. Thus far, compound specific carbon isotope analyses from the Austrian sections demonstrate a lack of significant changes in the sedimentary organic matter that would indicate a causal relationship between a peak in green algae and a synchronous δ^13^C_org_ excursion^30^, however, more work is needed to resolve the nature of CIEs associated with pronounced peaks in green algae across other end-Triassic sections. Nonetheless, green algal blooms appear to be a widespread phenomenon across end-Triassic sections highlighting a similar response to other episodes of biotic crisis^31^. The microbialites thus capture biological (blooms of green algae) and geochemical (stable isotopes) information associated with the end-Triassic mass extinction, informing a longstanding debate^15^,^18^ surrounding the environmental factors that contributed to their formation.

Two hypotheses, which are not mutually exclusive, have been invoked for the extensive development of Phanerozoic microbialites during times of metazoan biotic crisis: (1) opening of niches previously occupied by organisms affected by the extinction together with suppression of grazing/bioturbation^32^,^33^ and (2) changes in carbonate saturation state promoting rapid lithification and favorable geochemical conditions for microbialite formation and preservation^34^. The occurrence of the microbialites at the same level as the mass extinction^17^ along with the incredibly widespread distribution of the same growth phases over a distance of ~100 km^13^,^19^, indicates the microbialite constructors were able to achieve ecological dominance over a vast aerial extent due to an impoverished metazoan community. The widespread development of microbial mats during a post-extinction ‘dead zone’^17^, indicates that in the absence of extensive bioturbation, the mats were able to expand laterally. A rich component of sedimentary pyrite suggests the presence of sulfate reducing bacteria below the photosynthetic surface mat layers^35^, indicating a complex, and typical, microbial mat stratification.

The preservation of delicate features within the microbialite including the exceptional preservation of filamentous microfossils predominately within carbonate-rich laminae (Fig. 4B–D) indicates rapid calcification/carbonate supersaturation. Whether this supersaturation is linked to the events of the end-Triassic extinction or simply a local feature is less certain. The Triassic-Jurassic transition hosts several sharp rises in atmospheric *p*CO_2_^3^,^4^,^5^ induced by CAMP volcanism, which are superimposed on a greenhouse interval of high *p*CO_2_. Although a rapid *p*CO_2_ rise may have led to transient ocean acidification^27^, an increase in volcanic outgassing has been shown to ultimately result in a longer-term period of carbonate oversaturation regardless of the timescales of CO_2_ input^36^,^37^. Warming and *p*CO_2_ rise enhance weathering, increasing the saturation state of the oceans with respect to calcium carbonate and creating a preservation mechanism for benthic microbial communities that flourished in the absence of metazoan pressures. These weathering feedbacks, however, are understood to operate on timescales of 10s of kyr, meaning enhanced surface ocean supersaturation is not reached until 10s of kyr post-CO_2_ injection^37^. If the microbialites were deposited on timescales much more rapid than 10 kyr, then either: (a) the supersaturated conditions under which they formed are simply a local environmental feature (not related to global surface ocean supersaturation due to atmospheric CO_2_ injection) or (b) the entirety of the microbialite unit is deposited under global surface ocean enhanced saturation state 10s of kyr after the initial CO_2_ injection, meaning the excursion in δ^13^C_org_ recorded in the microbialites reflects a subsequent CO_2_ injection^4^,^5^, (*p*CO_2_ records suggest CAMP produced 3-4 pulses of CO_2_ addition), changes in organic matter composition, or both. Finally, it is worth noting that abundant ooids, indicative of enhanced carbonate saturation, are rare in the SW UK Triassic-Jurassic succession, but make an appearance in St Audrie’s Bay and Lavernock Point^21^ in strata directly correlative to the Upper Cotham Member microbialites. Therefore, while a metazoan post-extinction ‘dead zone’^17^ may have facilitated the development and ecological dominance of extensive microbial mats, their preservation as delicate microbial mat textures (Fig. 3B) and widespread microfossils (Figs 3C,D and 4) was a product of carbonate oversaturation. Thus, both hypotheses for the proliferation of microbial structures in the aftermath of Phanerozoic biotic crises fit the data from the SW UK.

The observations from the Triassic-Jurassic interval in the SW UK are similar to observations made across other times of biotic crisis in which widespread carbonate microbialite buildups occur in the immediate aftermath of extinction^33^. The Cotham Member contains a clear switch from predominantly skeletal carbonate in the lower Cotham Member to microbial carbonate in the Upper Cotham Member across the mass extinction interval^17^. A possible reason for the limited spatial and stratigraphic extent of the microbialites may have to do with the relatively isolated nature of the extensional basins that developed across the Tethyan realm during the initial rifting of Pangaea, many of which may have nonetheless retained a connection to normal marine waters. The subsequent transgression that followed deposition of the Cotham Member^21^ may have drowned the extensive benthic photosynthetic microbial communities, terminating growth of the microbialites; however the established warmer and accelerated hydrologic conditions that likely resulted from CAMP-induced higher *p*CO_2_ levels continued to favor carbonate deposition into the overlying Langport Member. If the supersaturation that promoted the formation of the Cotham Marble is related to a global weathering increase, we would expect other sections across the Triassic-Jurassic interval to contain extensive deposits of microbial and/or predominately non-skeletal carbonate in marine and terrestrial settings. While such deposits indeed occur in extensional basins immediately above CAMP basalt deposits^38^ and early Hettangian strata^39^, a potential association with the CAMP and the end-Triassic extinction requires further investigation.

The synchronous occurrence of extraordinarily widespread microbialites at the same level as (1) the mass extinction, (2) an acme of prasinophytes, (3) unusually preserved microbial fossils from nearby intertidal settings and (4) the I-CIE in δ^13^C_org_, highlights the non-local environmental mechanisms that led to Cotham Marble microbialite formation. As seen across other times of environmental crises associated with episodes of high *p*CO_2_, the microbialites reflect a shift in carbonate deposition from skeletal- to microbial-dominated successions across the end-Triassic. Given hypothesized constraints on the typical rate of microbialite growth, the microbialites may capture a high-resolution glimpse into the initiation of the I-CIE. The thin nature of the microbialite unit emphasizes the need for more high-resolution analyses and microfacies investigations across this critical geobiological transition in Earth history.

## Methods

Rock samples were collected from the Lilstock Formation of the SAB and LP sections as well as at various other Cotham Marble microbialite-bearing localities (Fig. 2A). Hand size samples were cut, polished, and scanned on a high-resolution scanner for mesoscopic analyses. Microfacies analyses were carried out via thin section light microscopy and scanning electron microscopy (SEM). Corresponding thin section billets were micro-drilled at ~cm-scale intervals for high-resolution stable isotope analyses of carbonate carbon and oxygen measured on an Elementar Americas Inc. (Micromass Ltd) Isoprime stable isotope ratio mass spectrometer (IRMS) at the University of Southern California. Sample aliquots of carbonate samples (~30 mg) were used for measurements of δ^13^C_org_ using an EA coupled to a Picarro Cavity Ring Down Spectrometer (G2121-i). About 30 mg of carbonate powder (per sample) was wetted with 60 mL of deionized water and acidified overnight in HCl fumes under a slight vacuum. Samples were then dried overnight and the carbon content was measured using the Picarro CO_2_ analyzer. The relative precision of the TOC measurements, based on control analysis of a lab reference standard (Sulfanilamide and L-Glutamic Acid USGS40), was better than 2%. Replicate and triplicate precision on sample runs was better than 5%.

## Additional Information

**How to cite this article**: Ibarra, Y. *et al*. A microbial carbonate response in synchrony with the end-Triassic mass extinction across the SW UK. *Sci. Rep.* **6**, 19808; doi: 10.1038/srep19808 (2016).

## Supplementary Material

Supplementary Information
