# Supplementary material for: A microbial carbonate response in synchrony with the end-Triassic mass extinction across the SW UK
Source: Sci Rep. 2016 Jan 27;6:19808. doi: 10.1038/srep19808 (PMC4728401; doi:10.1038/srep19808)
Supplement: Supplementary Information [file srep19808-s1.pdf]

A microbial carbonate response in synchrony with the end-Triassic mass extinction across the SW UK

Authors: Yadira Ibarra, Frank A. Corsetti, Sarah E. Greene, and David J. Bottjer

Supplementary Figures

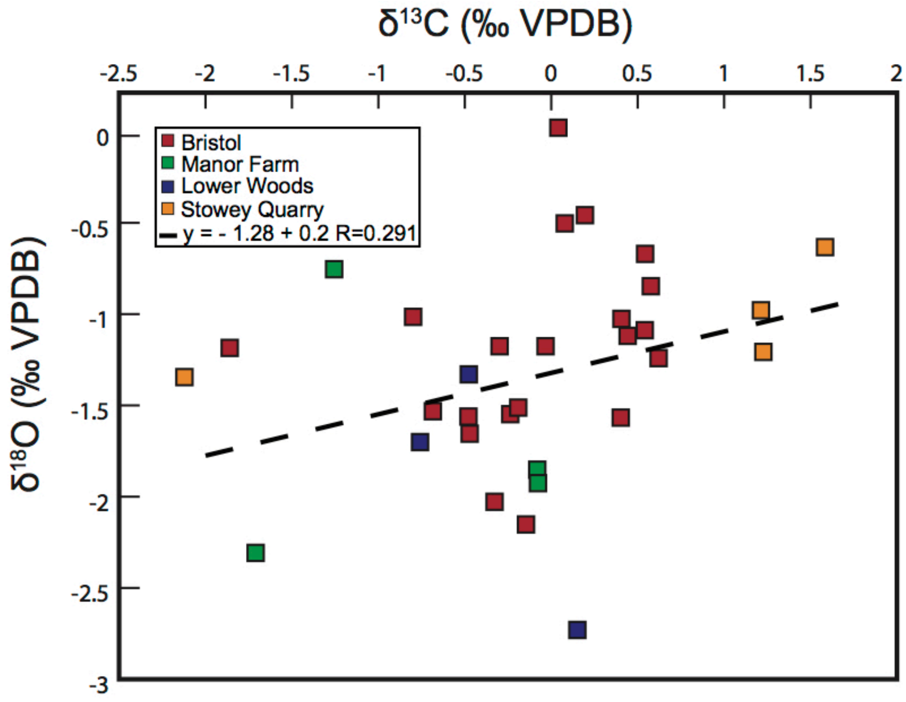

Figure S1. Cross plot of carbonate $\delta^{13}C$ and $\delta^{18}O$ of the Cotham Marble.

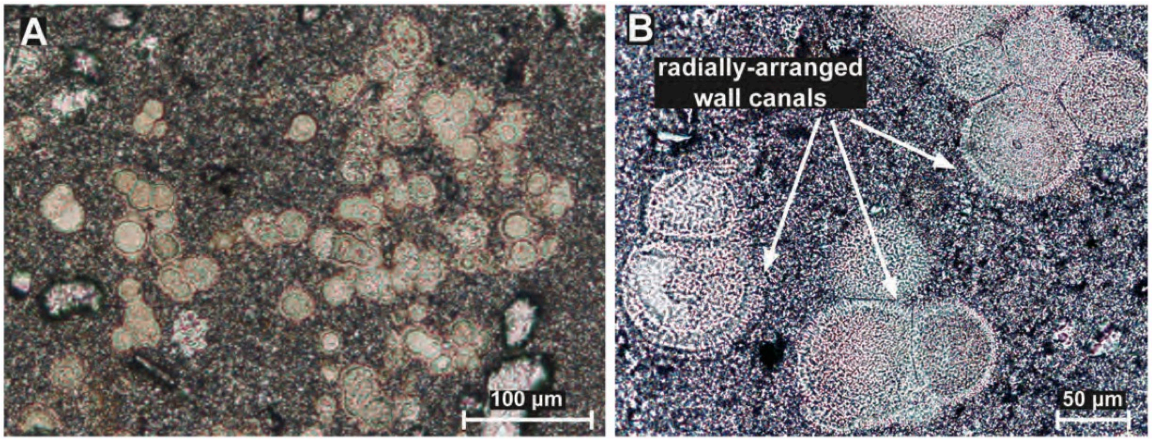

Figure S2. (A-B) Acme of prasinophyte phycomata assignable to *Tasmanites* from D1 of the Cotham Marble. Sample location: Manor Farm.

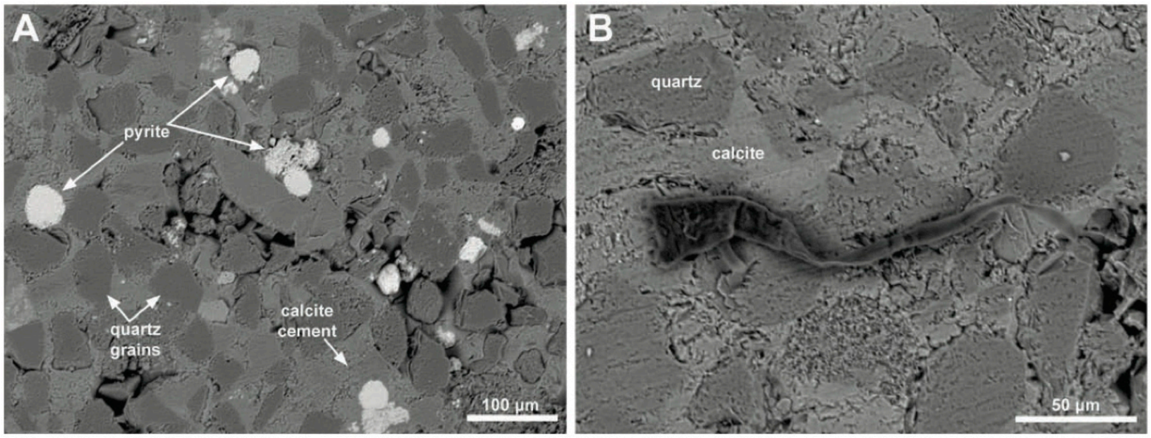

Figure S3. SEM photomicrograph of heterolithic facies of the Upper Cotham Member from Lavernock Point. (A) Pyrite grains embedded in a matrix of quartz of and calcite cement. (B) Filamentous microfossil in a quartz and calcite matrix.

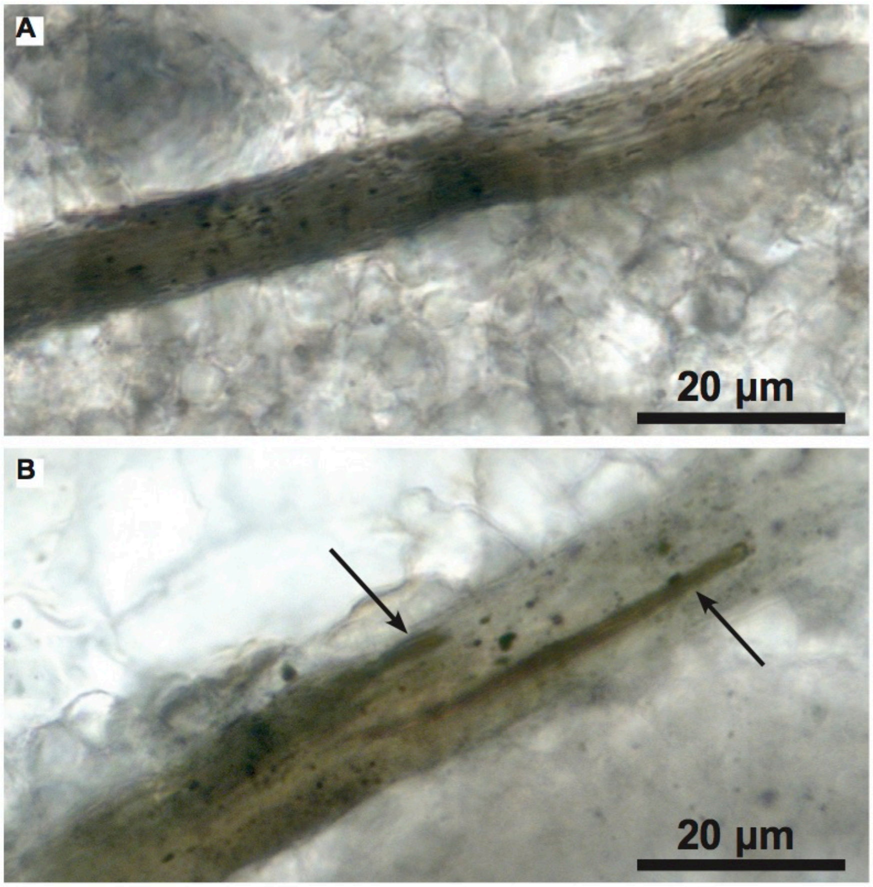

Figure S4. Thin section photomicrographs of filamentous microfossils. (A)-(B) Filamentous sheaths enclosing microbial filaments (arrows).

**Location**	**Sample ID**	**Layer**	**% Org C**	**$\delta^{13}C_{org}$**	**Avg. of duplicates/triplicates**
Manor Farm	MFD1s6	D1	0.16	-28.18	
Manor Farm	MFD1s6d	D1	0.18	-29.03	-28.61
Manor Farm	MFL1s2	L1	0.20	-27.03	
Manor Farm	MFL1s2d	L1	0.23	-27.89	-27.46
Manor Farm	MFL3	L3	0.18	-27.12	
Manor Farm	MFL3d	L3	0.20	-27.04	-27.08
Manor Farm	MFL2	L2		-27.02	
Manor Farm	MFD2f	D2		-27.65	
Manor Farm	MFD2d	D2		-27.34	
Manor Farm	MFDhed	D1	0.233	-28.15	
Manor Farm	MFDhedd	D1	0.237	-27.83	-27.99
Manor Farm	MFDL2	L2	0.199	-27.16	
Manor Farm	MFDL2d	L2	0.222	-27.18	-27.17
Pinhay Bay	RGL1	L1	0.21	-29.09	

Pinhay Bay	RGL1d	L1	0.22	-29.61	
Pinhay Bay	RGL1d2	L1	0.22	-29.29	-29.33
Pinhay Bay	RGD1	D1	0.23	-28.17	
Pinhay Bay	RGD1d	D1	0.22	-28.31	-28.24
Pinhay Bay	RGL2	L2	0.16	-27.45	
Pinhay Bay	RGL2d	L2	0.13	-27.75	-27.60
Bristol	BL3	L3	0.19	-28.55	
Bristol	BD1s	D1	0.22	-27.15	
Bristol	BD1sd	D1	0.21	-27.58	-27.37
Bristol	BD2cL1	L1		-26.71	
Bristol	BD2cL1d	L1		-27.28	-27.00
Bristol	CMBL2	L2		-26.75	
Bristol	BdL2	L2	0.196	-27.33	
Bristol	BdL2d	L2	0.202	-27.23	-27.28
Bristol	CMBD2f	D2		-28.60	
Bristol	CMBD2d	D2		-27.29	
Lower Woods	LWL2	L2	0.16	-27.11	
Lower Woods	LWdl	L1		-26.34	
Lower Woods	LWwl	L1		-25.96	
Lower Woods	LWf1	D1	0.179	-28.09	
Lower Woods	LWf1d	D1	0.188	-28.02	-28.06
Lower Woods	LWf2	D1	0.187	-27.93	
Lower Woods	LWf2d	D1	0.194	-28.07	-28.00
Lower Woods	LWf3	D1	0.199	-27.63	
Lower Woods	LWf3d	D1	0.180	-28.02	-27.82
Lower Woods	LWf4	D1	0.205	-27.67	
Lower Woods	LWf4d	D1	0.196	-28.21	-27.94
Lower Woods	LWf5	D1	0.186	-28.19	
Lower Woods	LWf5d	D1	0.196	-28.25	-28.22
Lower Woods	LWf6	D1	0.176	-28.44	
Lower Woods	LWf6d	D1	0.184	-28.80	-28.62
Lower Woods	LWD1hed	D1	0.239	-28.60	
Lower Woods	LWD1hed2	D1	0.230	-28.90	-28.75
Lower Woods	LWD2	D2	0.234	-26.86	
Lower Woods	LWD2d	D2	0.217	-27.34	-27.10
Lower Woods	LWD2hed	D2	0.237	-27.26	
Lower Woods	LWD2hedd	D2	0.222	-27.75	-27.51
Stowey Quarry		L1		-25.8	
Stowey Quarry		D1		-27.54	
Stowey Quarry		D1		-29.36	

Table S1. Stable isotopic composition of $\delta^{13}C_{org}$ (‰) of organic carbon of microbialite samples from their corresponding site and layer

Location	Layer	$\delta^{13}C_{carb}$	$\delta^{18}O_{carb}$
Bristol	L1	0.621	-1.241
Bristol	L1	-0.799	-1.015
Bristol	L2	-0.479	-1.561
Bristol	L3	0.576	-0.847
Bristol	D1	-0.237	-1.548
Bristol	D2	0.406	-1.026
Bristol	L1	0.442	-1.119
Bristol	L1	-0.033	-1.176
Bristol	D1	-0.146	-2.152
Bristol	D1	0.078	-0.503
Bristol	D1	-0.299	-1.176
Bristol	L2	0.402	-1.567
Bristol	L3	0.543	-0.670
Bristol	D2	-0.472	-1.655
Bristol	D2	-0.328	-2.028
Bristol	L1	0.195	-0.458
Bristol	L2	-0.685	-1.532
Bristol	L1	0.041	0.021
Bristol	L1	-1.860	-1.185
Bristol	D1	0.541	-1.088
Bristol	L3	-0.191	-1.512
Manor Farm	D1	-0.081	-1.852
Manor Farm	D1	-1.256	-0.754
Manor Farm	L2	-1.710	-2.308
Manor Farm	L1	-0.077	-1.926
Lower Woods	L1	-0.477	-1.330
Lower Woods	L1-d	-0.759	-1.701
Lower Woods	D1	0.151	-2.730
Stowey Quarry	L1	-2.122	-1.345
Stowey Quarry	D1	1.227	-1.207
Stowey Quarry	L2	1.584	-0.633
Stowey Quarry	L2-d	1.213	-0.979

Table S2. Stable isotopic compositions of $\delta^{18}O$ and $\delta^{13}C$ (‰ VPDB) for carbon and oxygen from their corresponding site and microbialite layer.
